# Accuracy of the WatchBP Office Central as a Type 2 device for non-invasive estimation of central aortic blood pressure in children and adolescents

**DOI:** 10.1038/s41371-024-00956-9

**Published:** 2024-09-13

**Authors:** Jonathan P. Glenning, Kieran Sandhu, Hilary A. Harrington, Lucas Eastaugh, Geoffrey K. Lane, Joseph J. Smolich, Jonathan P. Mynard

**Affiliations:** 1 Heart Research, Murdoch Children’s Research Institute, Parkville, VIC Australia; 2 Department of Paediatrics, University of Melbourne, Parkville, VIC Australia; 3 Melbourne Medical School, University of Melbourne, Parkville, VIC Australia; 4 Department of Cardiology, Royal Children’s Hospital, Parkville, VIC Australia; 5 Department of Biomedical Engineering, University of Melbourne, Parkville, VIC Australia

**Keywords:** Diagnosis, Hypertension, Preventive medicine

## Abstract

High blood pressure (BP) in childhood is a recognised precursor of elevated cardiovascular risk in adulthood. Brachial BP is normally used for clinical decision making, but central BP may be a better marker of pressure load on the heart. There is a paucity of validated non-invasive, automated devices for estimating central BP in children and adolescents. In this study, we compared the WatchBP Office Central (a Type 2 central pressure estimation device) against a high-fidelity micromanometer in the ascending aorta of anaesthetised patients undergoing clinically-indicated catheterisation (*n* = 15, age 4–16 years). As a secondary aim, central systolic BP (cSBP) was also compared to two non-invasive estimation methods in 34 awake patients undergoing routine cardiac MRI (age 10–18 years). WatchBP substantially overestimated cSBP compared to the intra-arterial gold-standard reference (26.1 ± 7.4 mmHg), and recruitment was terminated at *n* = 11 (included in the analysis) due to high statistical certainty that the device would not pass the validation criteria of 5±8 mmHg. WatchBP cSBP was also substantially higher than values obtained from a phase contrast MRI method (11.8 ± 7.9 mmHg) and the SphygmoCor XCEL (13.5 ± 8.9 mmHg) in the awake patient group, which translate to 21–23 mmHg on average after accounting for known/estimated biases in these non-invasive comparators. Compared with invasive central diastolic and systolic BPs, the brachial measures from WatchBP yielded errors of 0.1 ± 5.6 and 12.5 ± 6.0 mmHg respectively. We conclude that the WatchBP substantially overestimates cSBP in children and adolescents. These findings reinforce the need for central BP-measuring devices to be further developed and validated in this population.

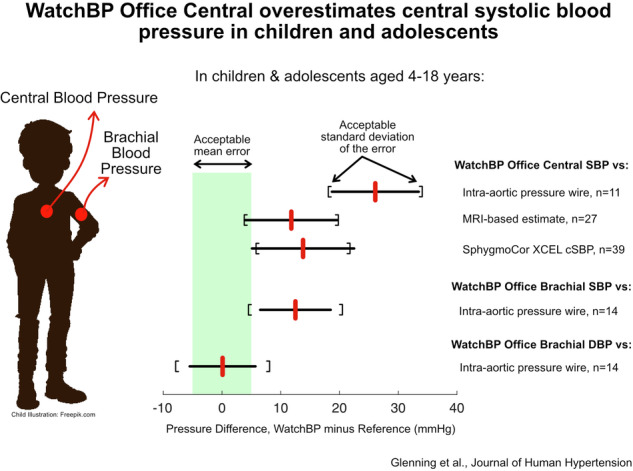

## Introduction

High blood pressure (BP) is the leading risk factor for disability and death across the world [1, 2], making its measurement a core component of clinical care. The deleterious effects of high BP, such as end organ-damage [3], can first become apparent in childhood, and likely underlie observed associations of high BP in childhood with fatal and non-fatal cardiovascular events in adulthood [4]. Although commonplace, measurement of a peripheral (i.e., brachial) systolic blood pressure (bSBP) overestimates central (i.e., aortic) systolic blood pressure (cSBP) due to pulse pressure amplification (PPA) [5–8]. PPA varies between individuals and depends on factors such as age, sex, height, and arterial stiffness [9, 10]. Thus, bSBP is not an entirely accurate measure of the pressure load facing the heart. Since PPA tends to be greater in young individuals with compliant arteries [11], assessment of cSBP is likely to be particularly important in children and adolescents.

For convenient estimation of cSBP, multiple devices utilising cuff-based plethysmography exist, using proprietary algorithms based on a transfer function and/or pulse wave analysis. Prior studies evaluating other devices, such as the SphygmoCor XCEL or Mobil-O-Graph [12, 13], against invasively measured aortic blood pressure found that these devices overestimated cSBP in children and adolescents, with errors in pulse calibration, arising from inaccuracies in the oscillometric measurement of bSBP, being the primary culprit [13]. It may therefore be expected that a device that provides accurate bSBP measurements would attain more satisfactory performance in estimating cSBP [13–19]. The WatchBP Office Central (Microlife AG, Widnau, Switzerland) utilises brachial pressure waveforms to estimate cSBP via an empirical equation. The device’s bSBP measurement has previously been validated in adults and children against the auscultatory method [20], and its cSBP algorithm passed validation against invasively measured blood pressures in adults [21]. However, the accuracy of cSBP provided by this device in children and adolescents is unknown.

The gold-standard method to evaluate cSBP devices is intra-arterial measurements with a high-fidelity micromanometer [22]. However, micromanometer devices are expensive and not routinely used for clinical purposes; in addition, in children and adolescents, invasive measures can only be ethically obtained during clinically indicated procedures under general anaesthesia. The ARTERY Society Taskforce suggested that ‘in future, it may be reasonable to use non-invasive central BP devices as reference standards, but the acceptance criteria for this are yet to be determined.’ Whilst this situation has not changed, in the present study we investigate a novel ‘method-fusion’ approach, whereby the primary analysis compares the test device against the gold-standard intra-arterial reference (under anaesthesia), but as a secondary analysis we also explore whether two non-invasive methods would (despite not being gold standard methods) support the generalisability of those results to the awake state.

The first non-invasive comparator is the SphygmoCor XCEL, a cuff device that was previously shown to overestimate intra-arterial cSBP by 7.9 mmHg in children and adolescents [13]. Given the relatively narrow 95% confidence interval of this difference (6.2–9.6 mmHg), we hypothesise that the difference between the test device and the XCEL device, plus 7.9 mmHg, would provide similar errors to the intra-arterial comparison.

Ascending aortic phase-contrast MRI (PCMRI) presents another possible non-invasive cSBP comparator using the technique described by Quail et al. [23]. This involves (1) extracting the aortic cross-sectional area waveform via segmentation of PCMRI magnitude images; (2) calibrating the waveform to brachial mean (MAP) and diastolic (bDBP) blood pressures, which are relatively similar to corresponding central values [24]; and (3) estimating cSBP as the peak value of the calibrated waveform.

In summary, the primary aim of this study was to evaluate the accuracy of cSBP as estimated by the WatchBP Office Central cuff device in children and adolescents, via comparison against high-fidelity intra-arterial measurements (during aortic catheterisation under anaesthesia). Given that anaesthesia results in relatively low blood pressure, a secondary aim was to provide corroborating data by comparing the test device with two non-invasive comparators (SphygmoCor XCEL and PCMRI) that can be applied in the awake state. Additional secondary aims were to evaluate (1) the agreement between bSBP from the test device and intra-arterial cSBP, (2) agreement between test device bSBP and an estimated intra-arterial bSBP obtained from central pressures and brachial applanation tonometry, and (3) the accuracy of test device DBP against intra-arterial measurements.

## Methods

The study was conducted in accordance with the Declaration of Helsinki and was approved by the Human Research Ethics Committee of the Royal Children’s Hospital, Melbourne, Australia (RCH). Written informed consent was obtained from parent(s)/guardian(s), or from the participants themselves when appropriate.

### Test device

The Watch BP Office Central (Microlife AG, Widnau, Switzerland) is a brachial cuff device that estimates cSBP as follows: (1) bSBP and bDBP are measured via oscillometry during smooth deflation at approximately 3.5–4.0 mmHg/s (based on our recordings); (2) The device then holds cuff pressure at 60 mmHg and performs volume plethysmography to obtain the pulse waveform, which is then automatically calibrated to bSBP and bDBP; (3) Various features (area under the diastolic and systolic parts of the waveform, late systolic shoulder, and end-systolic pressure) are extracted from the waveform and entered into an empirical multivariable equation to estimate cSBP [25]. As the device claims to estimate absolute intra-arterial aortic pressure, it may be classified as a Type 2 device according to the ARTERY Society Taskforce Consensus Statement [22].

### Study design

To assess the accuracy of the test device, we performed two studies. In Study 1 (primary analysis), we compared the test device against invasive high-fidelity blood pressure obtained during routine invasive cardiac procedures. In Study 2 (secondary analysis), we additionally compared the test device against two non-invasive comparators for cSBP. These studies obtained data from two patient groups (Group 1 and 2) which are detailed below; the intra-arterial comparison was conducted in Group 1 only, whilst non-invasive comparisons were conducted in one or both of Groups 1 and 2.

### Study 1—comparison with high-fidelity intra-arterial catheter

Details of blood pressure measurement during cardiac catheterisation procedures are similar to those described previously [13]. Briefly, 15 patients aged 4–16 years scheduled for a clinically-indicated cardiac catheterisation under general anaesthesia at RCH were recruited (Group 1). Invasive pressure data from four participants were excluded from analysis due to instrumentation and data recording problems. After the clinical procedure was complete, and while patients were still anaesthetized, a single-use high-fidelity micromanometer-tipped Verrata® Pressure Guide Wire (Volcano, CA, USA) was advanced to the tip of a previously positioned catheter located in the ascending aorta and was interfaced with a ComboMap system and PowerLab data acquisition system (AD Instruments, Dunedin, New Zealand). The mean value of the pressure signal recorded by the wire was calibrated to the mean value of the pressure signal obtained from the fluid-filled catheter, and signals were recorded continuously and simultaneously during measurements with the cuff devices. Each child’s arm circumference was measured, and they were fitted with appropriately sized cuffs based on the manufacturer’s guidance (Small cuff: arm circumference range 14–22 cm, bladder size 10 × 15.5 cm; M-L cuff: arm circumference range 22–42 cm, bladder size 13 × 23 cm). At least two recordings were taken on the left arm with the WatchBP Office Central (Serial number prefix: 2018-09-21, default software), simultaneously with intra-arterial recordings.

After removing the cuff, applanation tonometry was performed on the brachial artery to obtain a high-fidelity peripheral pulse waveform. As in our prior study [13], reference intra-arterial brachial systolic blood pressure (bSBP) was then estimated from the tonometric waveform after calibration to intra-aortic mean and diastolic pressures, which differ little between central and brachial sites [24]. Accordingly, we also compared the bDBP from the test device against centrally measured intra-aortic diastolic blood pressure (cDBP), noting that the test device does not report a separate central diastolic pressure.

### Study 2—comparison with other non-invasive central pressure estimation methods

Whilst the intra-arterial comparison forms the primary analysis of this study, as it is a gold-standard reference, we also compared the test device against two other non-invasive central pressure estimation methods: namely PCMRI and the SphygmoCor XCEL (AtCor Medical, Sydney, Australia).

### PCMRI

The PCMRI method was described by Quail et al. [23]. Although not validated against an invasive reference, the method has the advantage that it directly assesses the individual’s central aortic pulse waveform; if accurately calibrated to brachial mean and diastolic blood pressures, this pulse waveform can be used to derive cSBP. An advantage for the paediatric population is that the method can be applied in the unanaesthetised state.

Thirty-four patients aged 10–18 years undergoing a routine PCMRI without anaesthesia at RCH were recruited (Group 2). At least two recordings were taken with the WatchBP Office Central on the right arm in the rested, supine position in a holding bay next to the MRI room. Shortly thereafter, as part of the clinical protocol, each participant then underwent a through-plane PCMRI scan of the ascending aorta at the level of the right pulmonary artery, performed by an Aera 1.5 Tesla MRI machine (Siemens, Erlangen, Germany). A calculated temporal resolution of 128 phases per cardiac cycle was achieved through interleaved sampling of the two segments acquired per heartbeat, which was subsequently interpolated to 256 phases.

Cross-sectional area (*A*) waveforms were obtained from PCMRI via semi-automated segmentation of the aortic blood-wall boundary, using a custom in-house program written in MATLAB (R2020b, The MathWorks Inc., Natick, Massachusetts) [26]. The resulting waveforms were then calibrated using a linear two-point calibration, with the mean and minimum area mapped to brachial mean arterial pressure (MAP) and bDBP, respectively. bDBP was obtained directly from WatchBP, while MAP values were calculated from WatchBP bSBP and bDBP using the equation: (bSBP × 0.39) + (bDBP × 0.61), where the factor 0.39 is based on tonometric brachial waveform measurements in children and adolescents from a prior study in a similar patient group [13]. Note that the MAP and bDBP used for the PCMRI method were obtained from the test device itself, which tend to bias results in favour of the test device. We therefore also calculated a ‘corrected bias’ that accounted for the known error in bSBP used for calibration, based on the brachial tonometry data in Study 1, which indirectly estimates the average difference between the test device and intra-arterial pressure in the awake state.

### SphygmoCor XCEL

The SphygmoCor XCEL was employed as a second non-invasive reference, noting that it is one of the most widely used cuff-based central pressure devices in the field. However, the accuracy of this device was previously evaluated against a high-fidelity intra-aortic catheter and was found to overestimate cSBP by 7.9 mmHg in 62 paediatric participants [13]. We therefore also calculated a corrected bias for this comparator by adding this known bias of 7.9 mmHg to the measured difference between the XCEL device and test device.

Two measurements from the XCEL device were performed immediately before or after the WatchBP measurements in both Groups 1 and 2.

### Statistics

The ARTERY Society Taskforce recommended a minimum sample size of 85, based on the American Academy for the Advancement of Medical Instrumentation (AAMI) standard [27]. However, due to the very large and consistent differences seen between the intra-arterial reference and the test device, it was determined that additional recruitment beyond *n* = 11 (included in the primary analysis) could not be justified; the statistical grounds for early termination due to futility are explained in the Discussion.

Statistical analyses were performed with Prism (Version 9.3; GraphPad Software, San Diego, CA, USA). Clinical characteristics and blood pressure are presented as mean ± standard deviation, or n (%). Data were found to be normally distributed using the Kolmogorov-Smirnov test for normality. Unpaired t-tests and chi-squared tests were used for comparison of patient characteristics between groups. Paired t-tests were used to compare central pressure values obtained from WatchBP and each of the reference techniques. A modified Bland-Altman plot was generated for each pressure comparison, with the reference pressure (rather than the mean of the reference and estimated pressures) presented on the x-axis [28]. For each comparison, the acceptance thresholds for accuracy and precision proposed by the AAMI were used, i.e. a mean difference of less than 5 mmHg, and a standard deviation of the difference of less than 8 mmHg [27]. Furthermore, blood pressure values from individual recordings were used for statistical comparisons rather than participant averages, in accordance with guidance on statistical and reporting standards for validation studies by Stergiou et al. [29]. Similar results were obtained if intra-individual values were averaged before analysis.

## Results

The characteristics of the participants in Groups 1 and 2 are detailed in Table 1. Group 1 was younger, accounting for the lower height, weight, body surface area and arm circumference compared with Group 2. This in turn, along with the effects of general anaesthesia, likely contributed to the lower brachial blood pressures in Group 1 than Group 2. In Group 1, three participants had no usable WatchBP cSBP readings due to an unknown cause; all recorded Error E12, suggestive of ‘movement or muscle tension’ according to the device manual, despite the patient being anaesthetised and immobile, and three participants did not have SphygmoCor XCEL cSBP readings due to diastolic pressure being lower than 40 mmHg (no cSBP estimate is provided in this range). There were no failed readings by WatchBP in Group 2, although seven participants did not have a high temporal resolution PCMRI performed (a decision of the cardiologist for clinical reasons) or the PCMRI was not of sufficient quality due to motion artefact, and seven participants did not have SphygmoCor XCEL measurements taken due to time constraints.

**Table 1:** Clinical characteristics of participants.

Variable	Group 1	Group 2
Age (years)	8.1 ± 4.1	15.6 ± 2.6**
Sex (female)	8 (62)	15 (44)
Height (cm)	124.1 ± 26.8	165.4 ± 13.5**
Weight (kg)	32.3 ± 25.1	59.0 ± 17.1**
Body surface area (m^2^)	1.0 ± 0.5	1.6 ± 0.3*
Arm circumference (cm)	20.4 ± 5.6	25.4 ± 3.9**
Heart rate (bpm)	80 ± 9	70 ± 11*
Brachial systolic pressure (mmHg)	91.6 ± 11.1	115.9 ± 12.1**
Brachial diastolic pressure (mmHg)	48.4 ± 8.4	65.6 ± 6.2**
ASD/PDA/PFO/VSD	7 (47)	7 (21)
Transposition of the Great Arteries	3 (20)	7 (21)
Tetralogy of Fallot	0 (0)	9 (27)
Controls (normal, healthy participants)	0 (0)	6 (18)
Coarctation of the Aorta	0 (0)	4 (12)
Pre-Fontan (BCPC)	4 (27)	0 (0)
Cardiomyopathy	1 (7)	2 (6)
Pulmonary atresia	0 (0)	3 (9)
Fontan	2 (13)	0 (0)
Pulmonary artery stenosis	0 (0)	2 (6)
Transplant (CM)	1 (7)	0 (0)

Data presented as mean ± standard deviation or n (%). Some participants had more than one condition; therefore, the tally of cardiac conditions is greater than the number of participants. There are 15 participants in Group 1 and 34 participants in Group 2. Three participants did not have an arm circumference recorded (therefore *n* = 46 for this comparison). The ‘Controls’ group refers to participants without a history of cardiac surgery and who were screened but found to have normal cardiac and vascular anatomy and function. The ‘Cardiomyopathy’ group refers to the following conditions: constrictive pericarditis; arrhythmogenic right ventricular cardiomyopathy; and myocarditis.

*ASD* atrial septal defect, *PDA* patent ductus arteriosus, *PFO* patent foramen ovale, *VSD* ventricular septal defect, *BCPC* bi-directional cavo-pulmonary connection, *CM* cardiomyopathy.

*p = 0.01 and **p ≤ 0.001 when Groups 1 and 2 were compared.

### Study 1—comparison with intra-arterial micromanometer

**Fig. 1:**
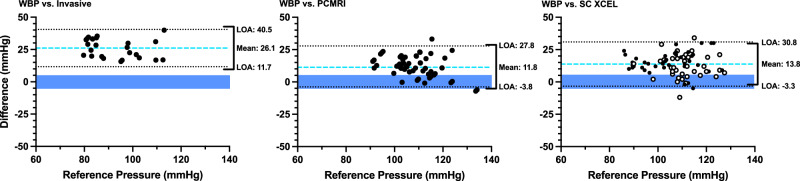
Modified Bland–Altman plots of WatchBP central systolic pressure compared against multiple reference methods. Plots compare the difference between the cSBP estimates of the WatchBP and the reference cSBP values (Y-axis) against the reference cSBP (X-axis). Bias (blue dashed lines) and limits of agreement (black dotted lines) are shown. *n* = 11 patients with 25 individual comparisons for the left panel, *n* = 27 with 57 comparisons for the middle panel, and *n* = 39 with 78 comparisons for the right panel. The blue shaded box represents the range of acceptable bias values. The black bracket on the right-hand side of each plot represents the acceptable limits of agreement (LOA, given the measured bias). In the third panel, the filled black dots represent data from data from Group 1 and the unfilled black dots represent data from Group 2.

The test device substantially overestimated cSBP when compared to the high-fidelity invasive reference (difference of 26.1 ± 7.4 mmHg, *p* < 0.001; Table 2 and Fig. 1). bSBP from the test device also overestimated intra-arterial cSBP (by 12.5 ± 6.0 mmHg, *p* < 0.001) and the reference bSBP obtained from the brachial tonometric waveform calibrated to central mean and diastolic pressures (by 11.6 ± 5.8 mmHg, Table 2). bDBP from test device did not differ statistically from intra-arterial cDBP in Group 1 (difference of 0.1 ± 5.6 mmHg, *p* = 0.96).

**Table 2:** Comparisons of WatchBP Office Central with the high-fidelity intra-arterial reference standard (Study 1).

	*n*	Comparisons	WatchBP	Reference	Difference	95% Confidence Interval
cSBP-WBP vs cSBP-IA	11	25	105.1 ± 10.7	79.0 ± 12.5*	26.1 ± 7.4	23.1–29.1
bSBP-WBP vs cSBP-IA	14	29	92.4 ± 10.6	79.9 ± 11.1*	12.5 ± 6.0	10.2–14.8
bSBP-WBP vs bSBP-Tx	11	11	90.3 ± 12.0	78.7 ± 11.7*	11.6 ± 5.8	7.7–15.6
bDBP-WBP vs cDBP-IA	14	29	49.1 ± 8.1	49.0 ± 10.0	0.1 ± 5.6	−2.1 to 2.2

Data presented as mean ± standard deviation. Pressures are in mmHg. Comparisons refer to the number of individual recordings being compared. There were three fewer participants for the cSBP-WBP comparison, as despite the test device providing an error for cSBP, it reported valid brachial pressures. Adequate tonometry waveforms could not be obtained in three participants.

*bDBP/cDBP* brachial/central diastolic blood pressure, *bSBP/cSBP* brachial/central systolic blood pressure, *IA* intra-arterial (ascending aorta), *Tx* obtained via the brachial tonometry waveform calibrated to intra-arterial (aortic) mean and diastolic pressures, *WBP* Watch BP (test device).

*p < 0.001 comparing WatchBP vs the reference method.

### Study 2—comparison with non-invasive methods

The WatchBP cSBP estimates were substantially higher than both the PCMRI-derived and SphygmoCor XCEL cSBP estimates (differences of 11.8 ± 7.9 and 13.8 ± 8.7 mmHg respectively, both *p* < 0.001; Table 3 and Fig. 1). There was no statistical difference between Group 1 (anaesthetised state) and Group 2 (awake state) for the XCEL vs test device comparison. Accounting for the estimated calibration error for the PCMRI method (based on the bSBP tonometry data in Table 2) and the known bias of 7.9 mmHg for the XCEL device [13] led to a corrected bias (i.e. average estimated cSBP vs intra-arterial difference) in the order of 21–23 mmHg in the awake state (Table 3).

**Table 3:** Comparisons of WatchBP with non-invasive comparators for cSBP (Study 2).

Method	*n*	Comparisons	Group	WatchBP	Reference	Difference	95% CI	Corrected bias
PCMRI	27	57	Group 2	114.3 ± 8.7	102.5 ± 11.8*	11.8 ± 7.9	9.7–13.9	23.4
XCEL	10	19	Group 1	105.9 ± 11.5	91.4 ± 12.0*	14.5 ± 8.2	10.6–18.5	22.4
XCEL	29	59	Group 2	117.2 ± 9.4	103.7 ± 9.1*	13.5 ± 8.9	11.2–15.8	21.4
XCEL	39	78	Group 1 and 2	114.5 ± 11.0	100.7 ± 11.1*	13.8 ± 8.7	11.8–15.7	21.7

Data presented as mean ± standard deviation. Pressures are in mmHg. Comparisons refer to the number of individual recordings. Corrected bias refers to the mean difference corrected for known or estimated biases in the respective non-invasive comparators.

*p < 0.001 comparing WatchBP vs the reference method.

## Discussion

This is the first study to have investigated the accuracy of the WatchBP Office Central device for estimating cSBP in children and adolescents. In the primary analysis (Study 1), WatchBP substantially overestimated cSBP compared to a high fidelity micromanometer placed in the ascending aorta, which is considered the gold-standard reference method [22]. Study 1 adhered to the ARTERY Society Taskforce Consensus Statement for validation of central blood pressure devices [22] (Supplemental Table 1), with the exception of sample size as the study was terminated due to futility (see statistical justification below). cSBP was also substantially overestimated when compared to two non-invasive methods, phase-contrast MRI, and the SphygmoCor XCEL (Study 2); although these are not gold-standard methods, they could be applied in the awake state and, after correction for known biases, provided strong indication that the invasive data (obtained under anaesthesia) were likely to be generalisable to the awake state. On the other hand, intra-arterial cDBP estimated by the WatchBP’s bDBP was well within the validation acceptance criteria (0.1 ± 5.6 mmHg), although this conclusion requires confirmation with a larger sample size.

The large overestimation of cSBP (26.1 mmHg) by WatchBP in children and adolescents, compared with a gold-standard high fidelity intra-arterial wire, was unexpected for multiple reasons. First, Cheng et al. [21] previously found that the WatchBP’s cSBP estimate in adults passed the accuracy criterion when compared to an invasive reference. Second, a previous study completed by our group, the KidCoreBP Study [13], found that for two other devices, SphygmoCor XCEL and Mobil-O-Graph, calibration error was the most likely source of error for the cuff-based cSBP estimates in children and adolescents. Given that the WatchBP has previously passed independent validation for the brachial pressure measurements in adults and children according to the ANSI/AAMI/ISO 81060-2:2013 standard [20], one may therefore have expected a lower calibration error and hence higher level of accuracy.

In addition to the invasive reference, Study 2 employed two other non-invasive methods for comparison with the WatchBP Office Central. The first, PCMRI, has the benefit that central pressure is estimated on the basis of subject-specific central aortic distension waveforms (a surrogate of the pressure pulse) and can be performed without sedation/general anaesthesia [23]. The significant overestimation of cSBP (11.3 ± 8.3 mmHg) by WatchBP compared with PCMRI-derived estimates occurred despite the fact that we used WatchBP’s brachial blood pressure measurements to calibrate the cross-sectional area waveforms, which would be expected to provide a favourable bias with respect to accuracy. Adding the error in bSBP estimated in Study 1 via brachial tonometry, the implied absolute cSBP error with respect to intra-arterial pressure was 23.4 mmHg, which suggests that the large overestimation observed in Study 1 was not a spurious finding caused by anaesthesia or the relatively low blood pressures in that physiological state.

SphygmoCor XCEL is one of the most widely used cSBP cuff devices and was the only reference method used in both Group 1 (anaesthetised) and Group 2 (awake). Both the KidCoreBP study [13] and the smaller study by Cai et al. [12] found that this device overestimated intra-arterial cSBP (with a bias of 7.9 and 13.8 mmHg respectively); hence, although this device should clearly not be considered a gold standard, the overestimation associated with this device may be considered well-established. If the WatchBP were accurate it would therefore be expected to produce lower values than those provided by the SphygmoCor XCEL [13]; instead, WatchBP values were 13.5 mmHg higher than those from SphygmoCor XCEL in Group 2 (awake state). Adding the known bias in the XCEL device implies that the test device overestimated intra-arterial cSBP by at least 21 mmHg in the awake state, which again supports the generalisability of the findings from Study 1.

The reason for this overestimation could be related to one or more of the following factors. First, an overestimation of brachial BP may be ‘transmitted’ to cSBP estimates in the form of pulse waveform calibration error. Kollias et al. [20] reported a small positive bias in bSBP for children and adolescents (1.6 mmHg) that could not account for the large errors seen in the present study, although the reference was auscultatory, rather than invasive measurements. By contrast, we estimated that bSBP was overestimated by 11.6 mmHg based on brachial tonometry and the intra-aortic measurements; however, this would only partially account for the error of >20 mmHg in cSBP. A second possible contributor to the cSBP overestimation is that the pulse waveform measured via volume plethysmography employed by WatchBP may result in pulse waveforms of insufficient quality, which translates to errors in cSBP. Moreover, whilst the WatchBP holds pressure at 60 mmHg to perform sub-diastolic plethysmography, this pressure is supra-diastolic in most of our patients; a more adaptive sub-diastolic pressure level may therefore be warranted in the paediatric setting. Finally, the empirical multivariate formula used by the WatchBP device to estimate cSBP, which involves 4 parameters derived from the pulse waveform and 5 coefficients [25], may not be valid in children and adolescents. Further work is needed to establish the source(s) of error, and how these could be remedied.

In contrast to cSBP, bDBP from WatchBP provided an acceptable estimate of intra-arterial cDBP (difference: 0.1 ± 5.6 mmHg, Table 3). Moreover, when considering bSBP from WatchBP, subtracting the bias of 12.5 mmHg from all WatchBP bSBP measurements would also yield cSBP estimates with acceptable accuracy and precision (0.0 ± 6.0 mmHg) compared with intra-arterial cSBP in this study. Although promising, both of these approaches would need to be validated in a larger, independent data set.

Several study limitations and methodological considerations should be noted. First, the sample size for Study 1 was considerably smaller than the 85 participants recommended by the ARTERY Society Taskforce. At least two statistical considerations justify the early termination due to futility (i.e. very low probability of a pass result if the study were continued). First, the 95% confidence interval for the mean difference was 23.1–29.1 mmHg, which is not remotely near the validation threshold. Second, using a simulation approach, we calculated that if the validation study were continued until *n* = 85 was reached, with three measurements in each individual, and these additional measurements reflected an exceptionally accurate device (0 ± 3 mmHg normally distributed differences), then when combined with the existing data, the final result would be 2.8 ± 8.5 mmHg. Hence in this extremely unlikely scenario, our conclusion that the device did not pass the validation criteria would be unchanged. The early termination of validation studies due to futility is not addressed in current validation protocols, but we suggest it should be considered in future.

Second, participants in Group 1 were anaesthetised and primarily children, while those in Group 2 were awake and predominantly adolescents. It is therefore noteworthy that comparisons of the test device vs SphygmoCor XCEL, which were available in both groups, were not statistically different between groups (Table 3). Moreover, the corrected biases for the non-invasive comparators were of a similar order of magnitude to those observed in the invasive study (21–23 mmHg vs 26 mmHg). Nevertheless, the somewhat higher error seen in Study 1 may be related to the lower blood pressures in Group 1, which were most likely caused by the younger age, higher proportion of females, and use of anaesthetic agents. Nevertheless, within Group 1, there was no significant correlation between blood pressure level and error (*p* = 0.18).

Finally, participants had a history of congenital disease or other clinical indications for catheterisation or MRI; however, patients with aortic obstructions or shunts at the time of measurement were excluded and therefore the arterial anatomy and haemodynamics were relatively normal.

## Conclusion

The WatchBP Office Central overestimated invasively measured cSBP in the anaesthetised state in children and adolescents, substantially exceeding accuracy criteria specified in validation guidelines. Data from two non-invasive comparators (PCMRI and SphygmoCor XCEL) strongly supported the generalisability of this result to the awake state. Although requiring confirmation in a larger sample, brachial WatchBP measures attained acceptable precision (with error standard deviations ≤6 mmHg) for estimating intra-arterial cSBP and cDBP, as well as acceptable accuracy for cDBP but overestimated invasively measured cSBP by 12.5 mmHg. In summary, the WatchBP did not pass validation as a Type 2 central pressure device in children and adolescents.

## Summary

### What is known about this topic

- Central blood pressure better quantifies ventricular afterload and stress on large arteries, compared with brachial blood pressure.
- There is a paucity of research investigating the accuracy of non-invasive central blood pressure estimation in children and adolescents.

### What this study adds

- This study showed that a commercial device for estimating central blood pressure (WatchBP Office Central) substantially overestimated central blood pressure when compared to high-fidelity invasive blood pressure in children and adolescents.
- WatchBP also substantially overestimated central blood pressure when compared to two other non-invasive comparators (SphygmoCor XCEL and phase-contrast MRI).
- The brachial blood pressures from WatchBP outperformed the estimated central blood pressure, suggesting that the empirical equation used to estimate central pressure in this device requires adaptation for use in children and adolescents.

## Supplementary information

Supplemental Material

## Data Availability

The data generated during this study are available upon publication to researchers who provide a methodologically sound proposal for use in achieving the goals of the approved proposal. Proposals should be submitted to data.requests@mcri.edu.au and will be subject to an ethical review process and approval.
