# Supplementary material for: Accuracy of the WatchBP Office Central as a Type 2 device for non-invasive estimation of central aortic blood pressure in children and adolescents
Source: J Hum Hypertens. 2024 Sep 13;38(12):814–20. doi: 10.1038/s41371-024-00956-9 (PMC11624132; doi:10.1038/s41371-024-00956-9)
Supplement: Supplementary file 1 — Supplemental Material [file 41371_2024_956_MOESM1_ESM.docx]

Supplemental Table 1 – Adherence of this study to the ARTERY Society Taskforce Guidelines for the intra-arterial comparisons.

| **ARTERY Society Taskforce Guideline** | **Fulfilled?** | **Comments** |
| --- | --- | --- |
| *Issues in the assessment and reporting of central blood pressure (BP) monitors and recommendations** | | |
| Disparity of non-invasive central BP devices as to what is being measured | Yes | The manufacturer claims that the device estimates intra-arterial central BP (i.e. Type II device). |
| Calibration of peripheral artery signals using brachial cuff BP | Yes | Calibration is handled entirely by the device. |
| Disparity in validation standards | Yes | Gold-standard high-fidelity intra-arterial was employed as the reference standard. |
| Limitations in performing invasive validation studies | Yes | The ARTERY Taskforce noted that “in future, it may be reasonable to use non-invasive central BP devices as reference standards, but the acceptance criteria for this are yet to be determined.” We assessed several non-invasive comparators as a secondary aim to add to this evidence base. |
| *Study Setting*** | | |
| Isolated room without disturbing influences | Yes | Conducted in catheterisation laboratory under anaesthesia. |
| *Non-invasive central BP device measurement standards* | | |
| List manufacturer, model, software version,  operating principles, signal processing step/s, calibration processes | Yes | Manufacturer/Model: Microlife, WatchBP Office Central  Software version: Default (2018)  Operating Principle: Pulse wave obtained via volume plethysmography at cuff pressure of 60 mmHg, central pressure then estimated via an empirical multivariable equation (see ^25^).  Signal Processing steps: Unknown/proprietary  Calibration Processes: Brachial SBP/DBP.^25^ |
| Time for BP measures; time points of brachial BP and central BP; cuff deflation speed | Yes | Reference intra-arterial central BP measured simultaneously with WatchBP measurement. Intra-aorta pressure averaged over the exact period of pulse volume plethysmography.  Cuff deflation speed: 3.5-4.0 mmHg/s (predetermined by the WatchBP device software). |
| Define and use appropriate cuff size | Yes | Cuff size determined from arm circumference and based on manufacturer’s instructions. |
| Dimensions of inflatable bladder for all cuff sizes available; process to determine cuff size | Yes | Details provided in the manuscript. |
| Process of familiarization with equipment | Yes | All research staff underwent thorough familiarization process with the equipment. |
| Separate validation studies for additional or optional features or functions | N/A | Default device settings were used. |
| Process/s of quality control; process used to delineate acceptable quality; number of un-acceptable readings; reason/s for exclusion | Yes | No recordings were excluded, aside from instances where the device threw an error and did not provide a result (see Results section for details). |
| *Invasive (intra-arterial) central BP reference standard* | | |
| Micromanometer-tipped catheter used if minor inflection points to be identified | Yes | Verrata® Pressure Guide Wire (Volcano, CA, USA). |
| Full description of catheter; frequency response and handling procedures | Yes | See above. Frequency response testing not required as the guide wire is high fidelity. Guidewire offset calibrated to fluid-filled catheter system referenced to mid-thorax. |
| Performance comparison of fluid filled catheter with micromanometer-tipped catheter | N/A | Micromanometer-tipped catheter used throughout. |
| *Data acquisition at rest* | | |
| Period of undisturbed rest; medications used | Yes | Participants were anaesthetised. Details of anaesthetic agents were as described in previous study.^13^ |
| No talking. Free from acute haemodynamic interventions | Yes | Participants were anaesthetised. |
| Test device compared with reference over time-period matching the test device deflation cycle; recorded under stable conditions | Yes | Exact matching of reference/test device measurement periods. Recordings were taken under conditions of haemodynamic stability. |
| Complete description of protocol; time interval between test device and reference measures | Yes | See Methods. No time interval between test and reference measures. |
| *Data acquisition at BP intervention* | | |
| Haemodynamic change from resting state | N/A |  |
| Description of the intervention procedure | N/A |  |

Items refer to those in *Table 2 and **Table 3 of the ARTERY Society Taskforce paper.^22^
